# Pancreatic Expression database: a generic model for the organization, integration and mining of complex cancer datasets

**DOI:** 10.1186/1471-2164-8-439

**Published:** 2007-11-28

**Authors:** Claude Chelala, Stephan A Hahn, Hannah J Whiteman, Sayka Barry, Deepak Hariharan, Tomasz P Radon, Nicholas R Lemoine, Tatjana Crnogorac-Jurcevic

**Affiliations:** 1 Centre for Molecular Oncology, Institute of Cancer & CR-UK Clinical Centre, Barts & The London School of Medicine (QMUL), Charterhouse Square London EC1M 6BQ, UK; 2 Molecular GI-Onkologie (MGO), University of Bochum, Germany

## Abstract

**Background:**

Pancreatic cancer is the 5th leading cause of cancer death in both males and females. In recent years, a wealth of gene and protein expression studies have been published broadening our understanding of pancreatic cancer biology. Due to the explosive growth in publicly available data from multiple different sources it is becoming increasingly difficult for individual researchers to integrate these into their current research programmes. The Pancreatic Expression database, a generic web-based system, is aiming to close this gap by providing the research community with an open access tool, not only to mine currently available pancreatic cancer data sets but also to include their own data in the database.

**Description:**

Currently, the database holds 32 datasets comprising 7636 gene expression measurements extracted from 20 different published gene or protein expression studies from various pancreatic cancer types, pancreatic precursor lesions (PanINs) and chronic pancreatitis. The pancreatic data are stored in a data management system based on the BioMart technology alongside the human genome gene and protein annotations, sequence, homologue, SNP and antibody data. Interrogation of the database can be achieved through both a web-based query interface and through web services using combined criteria from pancreatic (disease stages, regulation, differential expression, expression, platform technology, publication) and/or public data (antibodies, genomic region, gene-related accessions, ontology, expression patterns, multi-species comparisons, protein data, SNPs). Thus, our database enables connections between otherwise disparate data sources and allows relatively simple navigation between all data types and annotations.

**Conclusion:**

The database structure and content provides a powerful and high-speed data-mining tool for cancer research. It can be used for target discovery i.e. of biomarkers from body fluids, identification and analysis of genes associated with the progression of cancer, cross-platform meta-analysis, SNP selection for pancreatic cancer association studies, cancer gene promoter analysis as well as mining cancer ontology information. The data model is generic and can be easily extended and applied to other types of cancer. The database is available online with no restrictions for the scientific community at .

## Background

Pancreatic ductal adenocarcinoma (PDAC) usually presents at an advanced stage so that surgical cure is rarely achieved and conventional chemotherapy and radiotherapy have little impact, resulting in a very low 5-year survival rate (0.5%–5%) [1]. Thus a number of laboratories have focused on studying the evolution of pancreatic cancer from its earliest stages (pancreatic intraepithelial neoplasias or PanINs), putting pancreatic cancer among the best studied tumour tissue types at the molecular level. Thus a wealth of information regarding mutated and aberrantly expressed genes, miRNAs and proteins is now available, not only significantly boosting our biological understanding of the disease but also helping to identify new (early) diagnostic and therapeutic targets. Unfortunately, the huge and still rising volume and diversity of public pancreatic datasets makes it increasingly difficult for researchers to integrate this information into their current research efforts. In this report, we describe a dedicated Pancreatic Expression database [2] aiming to overcome this restriction, and furthermore propose it as a generic model for the organization, integration and presentation of complex cancer research data. The model is designed to address various research problems, ranging from the specimen origin and type, through cancer development stages to expression patterns. By bringing complex profiling data together, the Pancreatic Expression database should enable scientists worldwide to perform a whole range of user-friendly queries, from deciphering the biological mechanisms underlying pancreatic disease to target discovery.

## Construction and Content

### Construction

The aim of the Pancreatic Expression database is to provide a comprehensive mining tool for large-scale genomic, transcriptomic and proteomic data sets. In order to achieve this, we designed a robust internal structure encompassing specific pre-defined modules (which can be found under the "Filters" section in the database) including "*pancreatic specimen/cell type*", "*pancreatic differential expression information*", "*genes differentially expressed in*" and "*genes expressed in*" modules. Our design enables uploading of any available (pancreatic) datasets that comply with the structure of the pre-defined modules. Each module contains a number of subcategories related to the module name, which are fundamental to store and retrieve user-defined sub-datasets from the database by setting filters to the specific subcategories within each module. The "*pancreatic specimen/cell type*" module covers categories such as normal (microdissected ductal cells (ND) or bulk normal pancreas (NP), acinar cells, islet cells, stromal cells and pancreatic stellate cells), and disease specimens from both exocrine (pancreatic intraepithelial neoplasias (PanIN-1A, PanIN-1B, PanIN-2, PanIN-3), chronic pancreatitis (CP), pancreatic adenocarcinoma (PDAC), intraductal papillary mucinous neoplasms (IPMN), mucinous cystic tumours and ampullary carcinoma) and endocrine (functioning and non-functioning tumours) origin. Moreover, pancreatic juice, plasma, urine, serum, and fine needle aspirates are included as additional options to further broaden future expansion of the database. The "*pancreatic differential expression information*" module provides information on direction of regulation (up- and down-regulation), fold-change, SAGE tag number and whether a gene or protein was found to be expressed only in pancreatic adenocarcinoma (PDAC) or in normal pancreas. The "*genes differentially expressed in*" module enables more defined selection of comparison methods such as pancreatic adenocarcinoma (PDAC) versus normal pancreas (bulk tissue or microdissected normal ductal cells), chronic pancreatitis (CP) versus normal pancreas (bulk tissue or microdissected normal ductal cells), chronic pancreatitis (CP) versus pancreatic adenocarcinoma (PDAC), pancreatic intraepithelial neoplasias (PanIN-1A, PanIN-1B, PanIN-2 or PanIN-3) versus normal pancreas (ND) or microdissected normal ductal cells), etc. The "*genes expressed in*" module lists the genes expressed in the tissue types defined in the pancreatic specimen/cell module, irrespective of their mode of regulation (whether they are differentially expressed or not). The "*platform technology*" module enables the selection of the technology used, such as Affymetrix arrays, cDNA arrays, Sanger human 10K cDNA arrays version 1.2.1, Sanger custom 5K1 cDNA arrays, Clontech Atlas Human Cancer cDNA Expression Array, SAGE, Agilent Human Genome CGH array, 2D PAGE, SELDI, etc. The data is stored in a data management system created using MySQL [3] and based on the open-source BioMart technology [4], a simple, federated query system designed specifically for use with large datasets. We imported the available Ensembl [5] human genome annotations (Ensembl release 41) for genes and proteins, SNP information, sequences, gene structure and multi-species data enabling the integration and annotation of heterogeneous pancreatic cancer data. In order to avoid integration and annotations errors, we used the pre-established Ensembl annotations and microarray probe set mapping. Ensembl links to UniProt/Swiss-Prot, RefSeq and UniProt/TrEMBL databases are made on the basis of sequence similarity. All other subsequent links are inferred from these mappings. Ensembl also establishes mappings to microarray probe set identifiers by matching probe set sequences to Ensembl transcripts [6]. We also integrated the antibody data from the Human Protein Atlas [7] based on Ensembl gene ID.

### Content

The Pancreatic Expression database currently contains 32 datasets from 20 different published sources, from 14 international laboratories encompassing 22 different platforms (Affymetrix GeneChip Human Full Length Array HuGeneFL, Affymetrix GeneChip Human Genome U95 Set (HG-U95A, HG-U95B, HG-U95C, HG-U95D, HG-U95E), Affymetrix GeneChip Human Genome U133 Array Set (HG-U133A, HG-U133B), 2D PAGE, cDNA arrays, SAGE, Operon oligo array version 2.0, Clontech Atlas Human Cancer cDNA Expression Array, immunohistochemistry, in situ hybridisation, Oligo array, MALDI, mass spectrometry, Sanger human 10K cDNA arrays version 1.2.1, Sanger custom 5K1 cDNA arrays, United Gene Technique Ltd, BD PowerBlot Western array and qRT-PCR) [8-27]. These initial datasets provide valuable information about 7636 gene expression measurements from a first-pass selection of relevant papers in the field of pancreatic research; however, the inclusion of additional relevant datasets will be a continuous and ongoing process. All the datasets were manually processed, checked for accuracy and consistency and loaded into our relational database alongside annotations from several public resources such as Ensembl, GO, dbSNP, UniProt and the Human protein atlas. Currently, several modules are present for which data are either not yet incorporated or not available (ICAT, iTRAQ), but these will be populated as it is our intention to continuously extend the current data content and cover all the existing modules as and when the data becomes available.

## Utility and Discussion

### Data access

**Figure 1:**
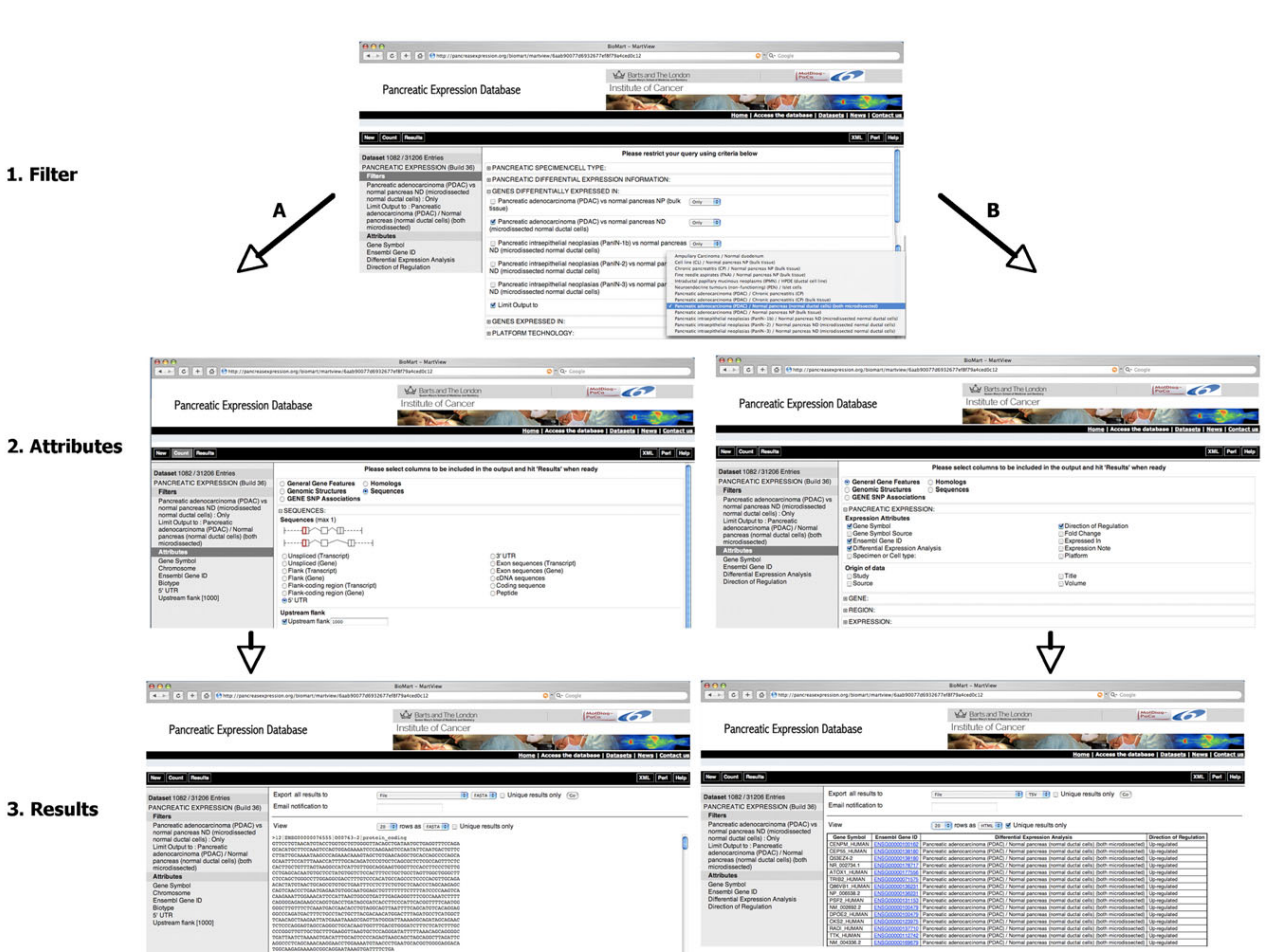
**The Pancreatic Expression database web-based query interface**. In this first example our goal was to identify differentially regulated genes in pancreatic cancer found in microdissected samples only and/or to extract the upstream sequence data for these genes. This query starts by choosing the "Pancreatic Expression Database" from the "*Choose Database*" drop-down selection in the right panel. Users will be automatically directed to the "*Pancreas Expression (Build 36*)" dataset with the left panel displaying the "*Filters*" and "*Attributes*" nodes with their default settings. The next step involves choosing the appropriate attributes and filters to restrict the query. Clicking on the "*Filters*" or "*Attributes*" nodes on the left will display the related page where "*Filters*" or "*Attributes*" are arranged into sections, which can be expanded/collapsed using the "+/-" box. To choose an attribute or a filter, users can simply click on the checkbox next to its description. A summary of the selected filters and attributes is automatically displayed in the left panel. Clicking on the "*Count*" button in the tool bar at any time when constructing the query will return the number of genes satisfying the pre-selected criteria. One can select the option to download the results to a file at the top of the result page and export them using the "GO" button. Again, there are options to change the format ("HTML", "CSV" for comma separated values, "TSV" for tab separated values, "XLS" for Excel, "ADF" for array description format) and whether to make the results unique. One can select a compressed file output and the query will run in the background to be downloaded later. One needs to provide an email address to receive an URL in a notification email that allows the query results to be downloaded. 1. Filter: in this example, the query was restricted by selecting the "*Filters*" node on the left and choosing the filter options on the right. Next, the filter section "*GENES DIFFERENTIALLY EXPRESSED IN:*" was expanded in order to select the "*Pancreatic adenocarcinoma (PDAC) vs normal pancreas ND (microdissected normal ductal cells*)" and limited the output to the "*Pancreatic adenocarcinoma (PDAC) vs normal pancreas ND (microdissected normal ductal cells*)" from the "*Limit Output to*" drop down menu. This is to avoid retrieving all the information about the genes satisfying the search criteria. 2. Attributes: these are arranged into five pages for "*General Gene Features*", "*Genomic Structures*", "*GENE SNP Associations*", "*Homologs*" and "*Sequences*". In order to select the output content, the "*Attributes*" node on the left needs to be clicked on and the attribute page on the right needs to be chosen. A. from the "*Sequences*" attribute page, the section "*SEQUENCES*" was expanded and "*5' UTR*" selected by typing in "*1000*" basepair as an "*Upstream flank*". B. from the "*General Gene Features*" attribute page, one can expand the attribute section "*PANCREATIC EXPRESSION*" where the "*Gene Symbol"*, "*Ensembl Gene ID*", "*Differential Expression Analysis*" and "*Direction of Regulation*" default options are displayed. 3. Results: clicking on the "Count" button displays 1082 genes satisfying the search criteria. Clicking on the "*Results*" button on the toolbar retrieves the final query results. By default, this shows the first 10 results in fasta format (A) and in hyper-linked HTML format (B) but the number and format can be altered using the drop-downs above this preview, as described above. Checking the "Unique results only" in (B) displays the unique rows only from the result table.

**Figure 2:**
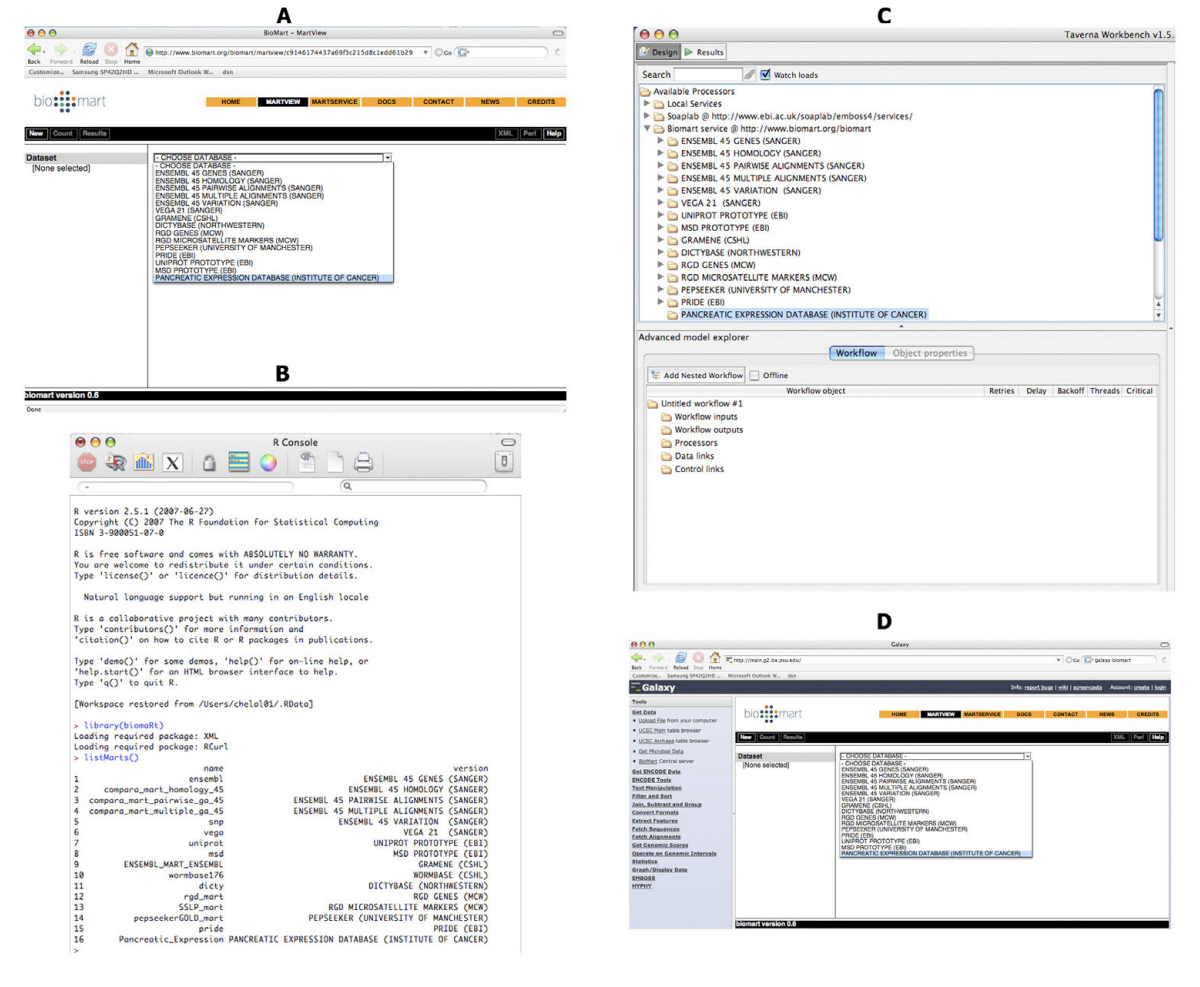
**Access through third party software**. The Pancreatic Expression database allows multiple levels of access. A – Access from the BioMart central server. B – Access within the open source R statistical environment through the biomaRt bioconductor package. C – Access from Taverna workflow system. D – Access with Galaxy.

**Figure 3:**
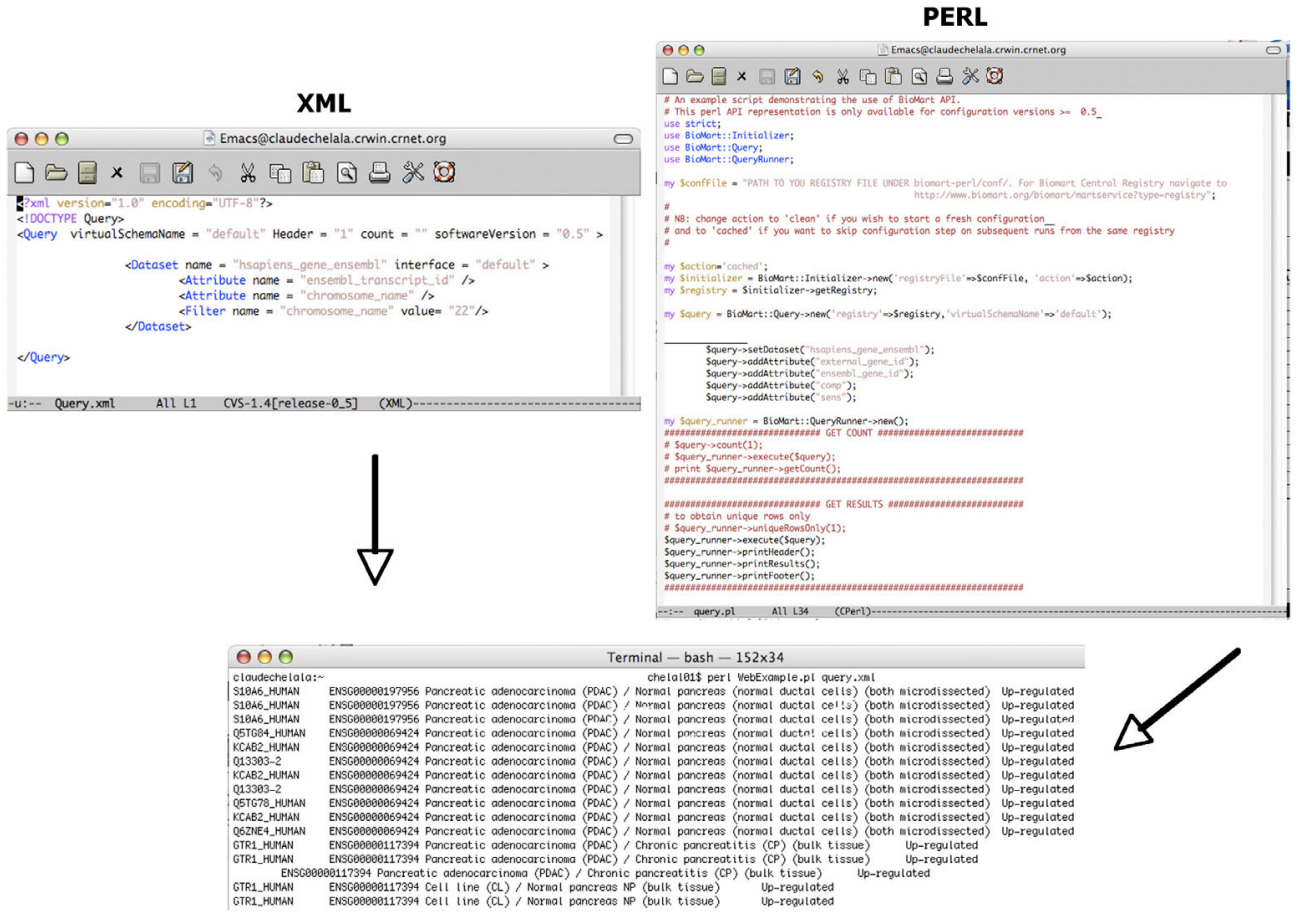
**Access provided programmatically through web services**. In this example we show how to submit a query using web services. One can generate an XML or Perl script by building up a query using the web-based user interface (as described in Figure 1) and hitting the XML or Perl button on the right side of the toolbar. One can save the query as query.xml or query.pl and then use it to query the database.

**Figure 4:**
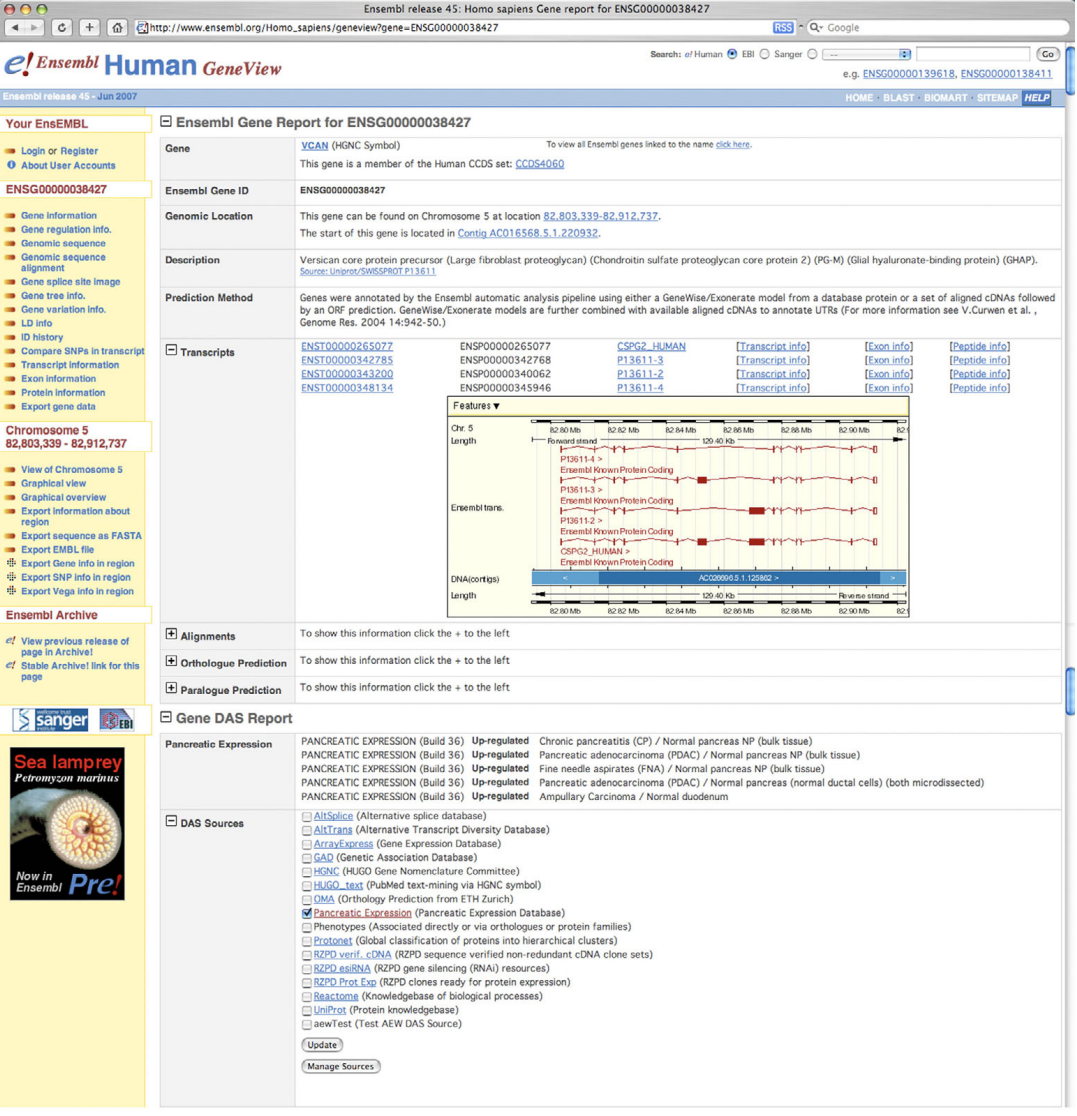
**The Pancreatic Expression DAS annotation available at the Ensembl GeneView**. The figure shows the Ensembl GeneView report for the ENSG00000038427 gene. The Pancreatic Expression database query results return Ensembl Gene ID by default with a hyper-link to the Ensembl GeneView website. In the Gene DAS Report section, which can be expanded/collapsed using the "+/-" box, a list of all the DAS sources including the Pancreatic Expression database is provided. To display the Pancreatic Expression database annotations, one can simply click on the checkbox next to the Pancreatic Expression and press the Update button at the end of the DAS sources.

The Pancreatic Expression database provides access not only to bioinformatic and biostatistic experts but also to bench researchers with a limited knowledge of bioinformatics. The database allows multiple levels of access. Firstly, access to the data is provided through a customized version of MartView, a BioMart web-based query interface based on Perl API [28]. The interface is navigated using the left panel with user selections taking place in the right panel. A summary of user choices is also displayed in the left panel. A simple query involves choosing attributes (or using the default ones) and optionally filters if one wants to restrict the query (Figure 1). Secondly, the Pancreatic Expression database is available from the BioMart central server (Figure 2A) where it is exposed to third party software such as the Bioconductor [29] package biomaRt [30] therefore allowing its easy interrogation within the open source R statistical environment [31] and its integration into any expression profiling experiment (Figure 2B). In addition, the database is exposed to the Taverna workflow system [32] (Figure 2C) and to the Galaxy framework (Figure 2D) [33]. The data can be also accessed programmatically through web services (Figure 3) [34]. A query constructed in the web-based query interface can be easily converted into an xml or perl template for future bioinformatics expansion and use. Finally, the Pancreatic Expression database is a DAS server providing a Pancreatic Expression DAS annotation available at the Ensembl GeneView (Figure 4) [5].

### Examples of use

Navigation between all data types is simple and user-friendly; a variety of possible query combinations allow researchers quickly to determine the most de-regulated genes and proteins across all platforms.

**Figure 5:**
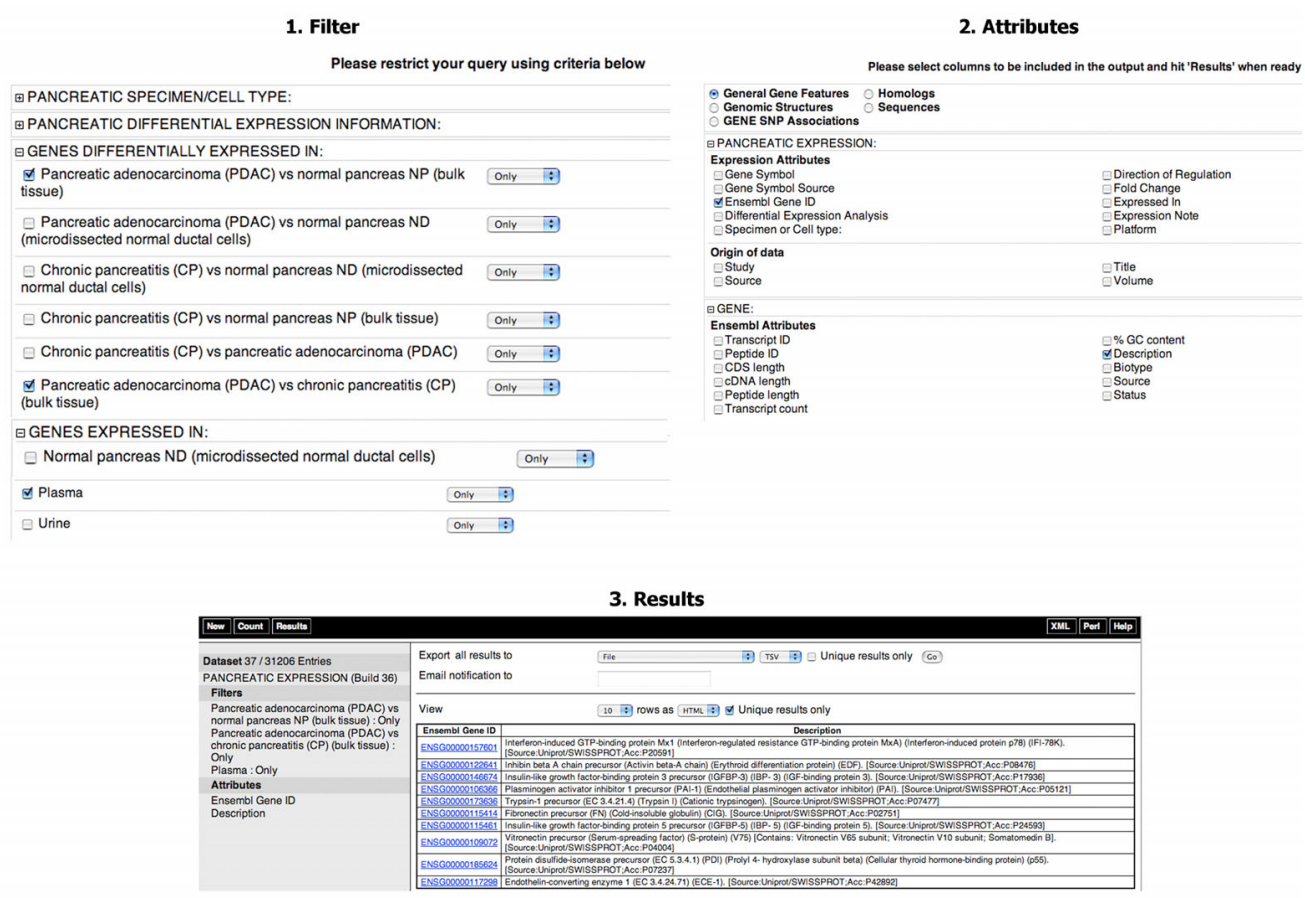
**Biomarker discovery**. The goal of this example was to retrieve genes expressed only in pancreatic cancer, not expressed in chronic pancreatitis and present in plasma. 1. Filter: the query is restricted by clicking on the "*Filters*" node on the left, expanding the filter section "*GENES DIFFERENTIALLY EXPRESSED IN:*" and selecting for genes differentially expressed in "*Pancreatic adenocarcinoma (PDAC) vs normal pancreas NP (bulk tissue*)" as well as "*Pancreatic adenocarcinoma (PDAC) vs chronic pancreatitis (bulk tissue*)" and expanding the filter section "*GENES EXPRESSED IN:*" and selecting for genes expressed in "*Plasma*". 2. Attributes: selected by clicking on the "*Attributes*" node on the left, choosing the "*General Gene Features*" attribute page, expanding the attribute section "*PANCREATIC EXPRESSION*" to remove "*Gene Symbol"*, "*Differential Expression Analysis*" and "*Direction of Regulation*" default options, expanding the attribute section "*GENE*" and selecting "*Description*" under "*Ensembl Attributes*". 3. Results: Clicking the "*Count*" button in the tool bar returns 37 genes satisfying the filters criteria. Clicking the "*Results*" button on the toolbar will retrieve a result table. Checking the "Unique results only" will display only the unique rows of the result table.

Using the Pancreatic Expression database, it is possible to search and retrieve genes/proteins expressed only in pancreatic cancer and not in chronic pancreatitis and then ask which of these are present in urine and/or plasma. Such a query would be a first step for the discovery of non-invasive pancreatic cancer biomarkers from body fluids (Figure 5).

**Figure 6:**
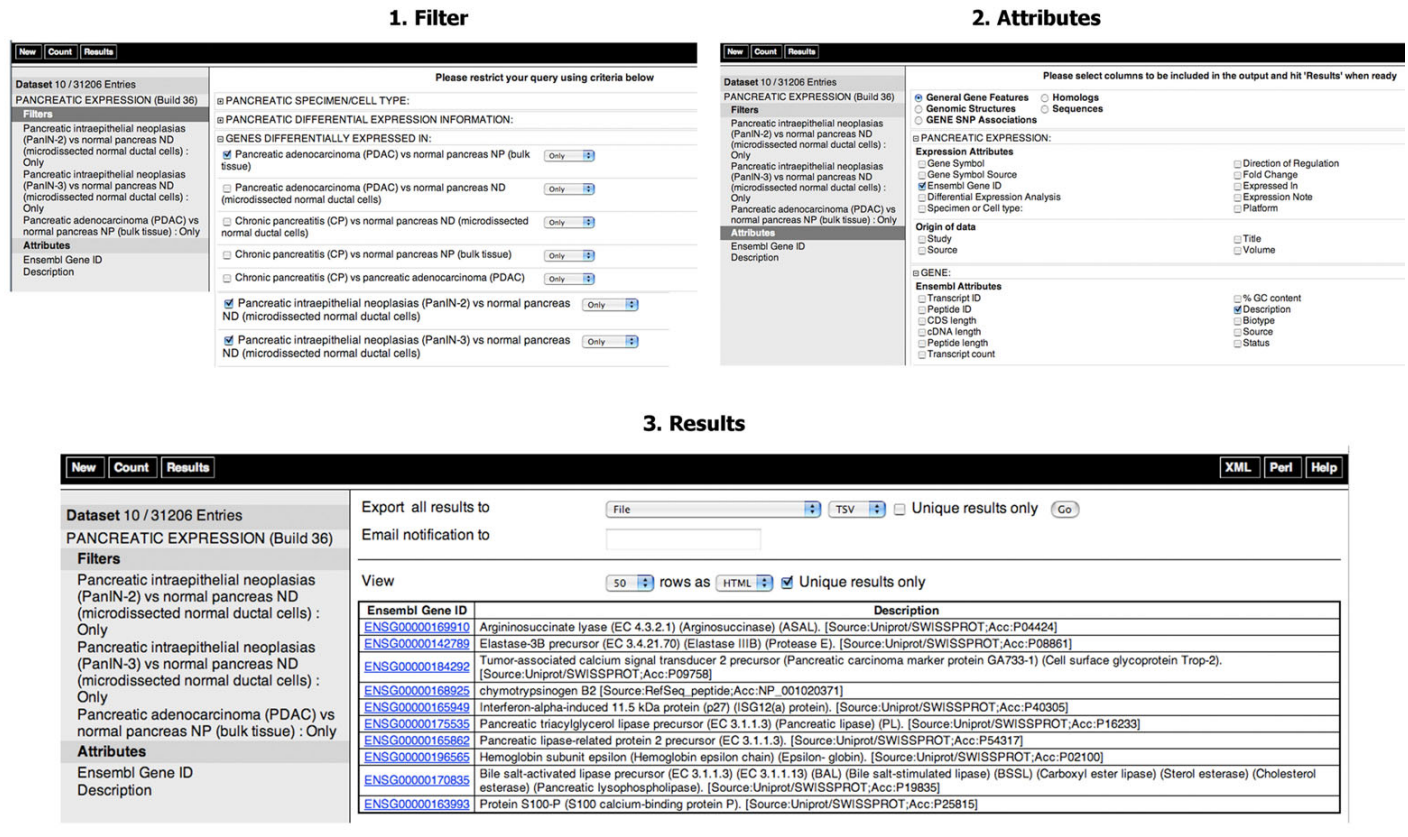
**Genes involved in the progression of pancreatic cancer**. The goal of this example was to query the differentially expressed information for pancreatic tumour stages (pancreatic intraepithelial neoplasias and pancreatic adenocarcinoma) and to retrieve the genes found to be de-regulated in the progression of pancreatic cancer. 1. Filter: one can choose "*Pancreatic intraepithelial neoplasias (PanIN-2) vs normal pancreas ND (microdissected normal ductal cells*)", "*Pancreatic intraepithelial neoplasias (PanIN-3) vs normal pancreas ND (microdissected normal ductal cells*)" and "*Pancreatic adenocarcinoma (PDAC) vs normal pancreas NP (bulk tissu*e)" from the filter section "*GENES DIFFERENTIALLY EXPRESSED IN:*". 2. Attributes: one can choose the "*General Gene Features*" attribute page, expand the attribute section "*PANCREATIC EXPRESSION*" to remove "*Gene Symbol"*, "*Differential Expression Analysis*" and "*Direction of Regulation*" default options, expand the attribute section "*GENE*" and select "*Description*" under "*Ensembl Attributes*". 3. Results: Clicking the "*Count*" button in the tool bar returns 10 genes involved in the progression of pancreatic cancer.

Researchers interested in the genes involved in the progression of pancreatic cancer can select the corresponding information among the differential expression datasets for the various tumour stages and retrieve the genes found to be de-regulated in the progression of pancreatic cancer (Figure 6). In the same way, one can search for genes specific to certain types of pancreatic cancer (Figure 1B).

**Figure 7:**
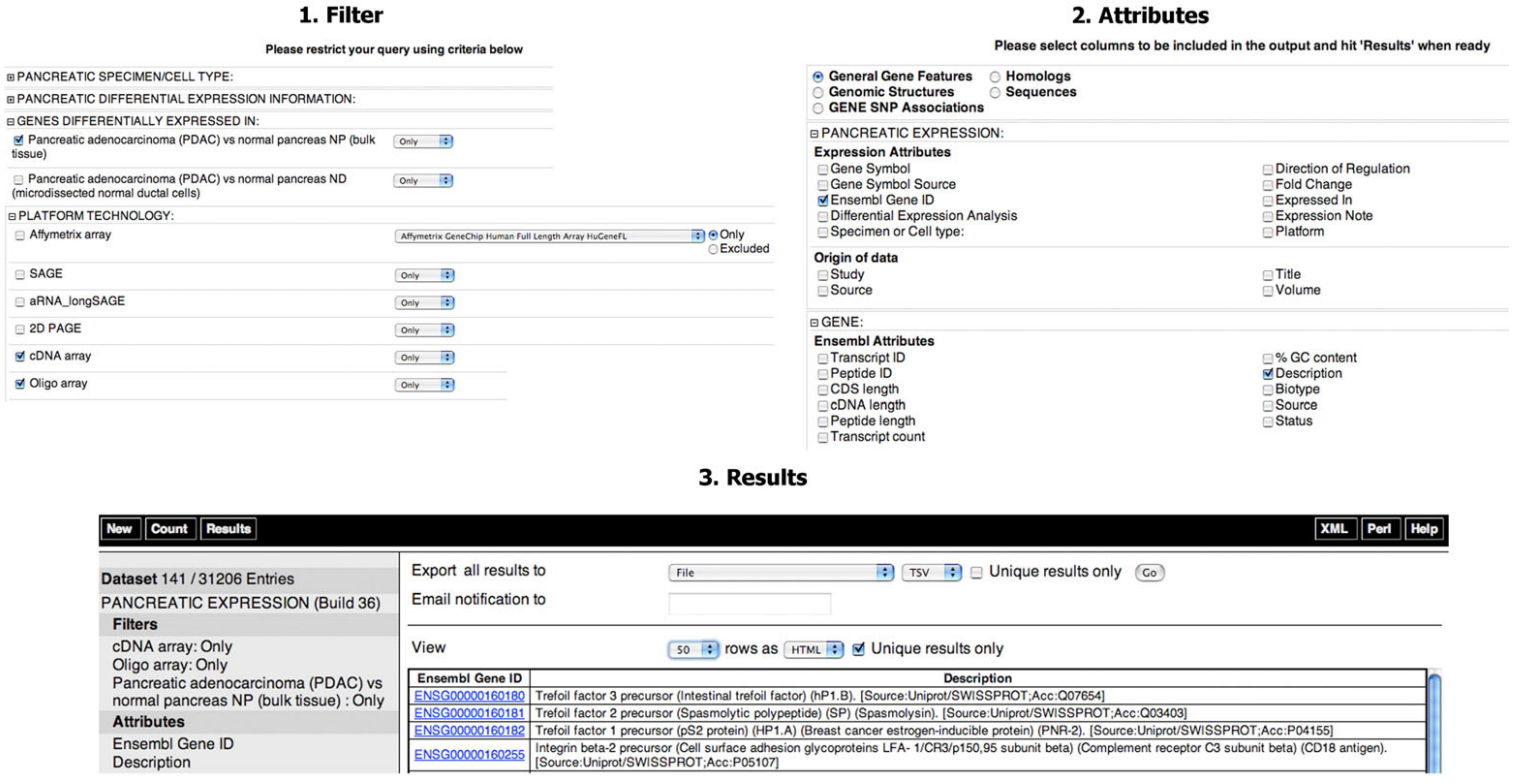
**Cross-platform analysis of pancreatic cancer data in the database**. The goal of this example was to investigate pancreatic expression profiling data obtained using cDNA and oligo arrays and to retrieve the common set of de-regulated genes. One can proceed as follows: 1. Filters: restrict the query by clicking on the "*Filters*" node on the left. Choose the cancer subtype, for example by expanding the filter section "*GENES DIFFERENTIALLY EXPRESSED IN:*" and selecting for genes differentially expressed in "*Pancreatic adenocarcinoma (PDAC) vs normal pancreas NP (bulk tissue*)", expanding the filter section "*PLATFORM TECHNOLOGY:" *and selecting both "*Oligo array*" and "*cDNA array*". 2. Attributes: select attributes by clicking on the "*Attributes*" node on the left, choosing the "*General Gene Features*" attribute page, expanding the attribute section "*PANCREATIC EXPRESSION*" to remove "*Gene Symbol"*, "*Differential Expression Analysis*" and "*Direction of Regulation*" default options, expanding the attribute section "*GENE*" and selecting "*Description*" under "*Ensembl Attributes*". 3. Results: hitting the "*Count*" button retrieves 141 de-regulated genes in cancer when using both oligo and cDNA platforms. One can proceed to saving the whole result set by choosing the appropriate action(s) at the top of the page, for example in an Excel format, and clicking the "*Go*" button to export the results.

**Figure 8:**
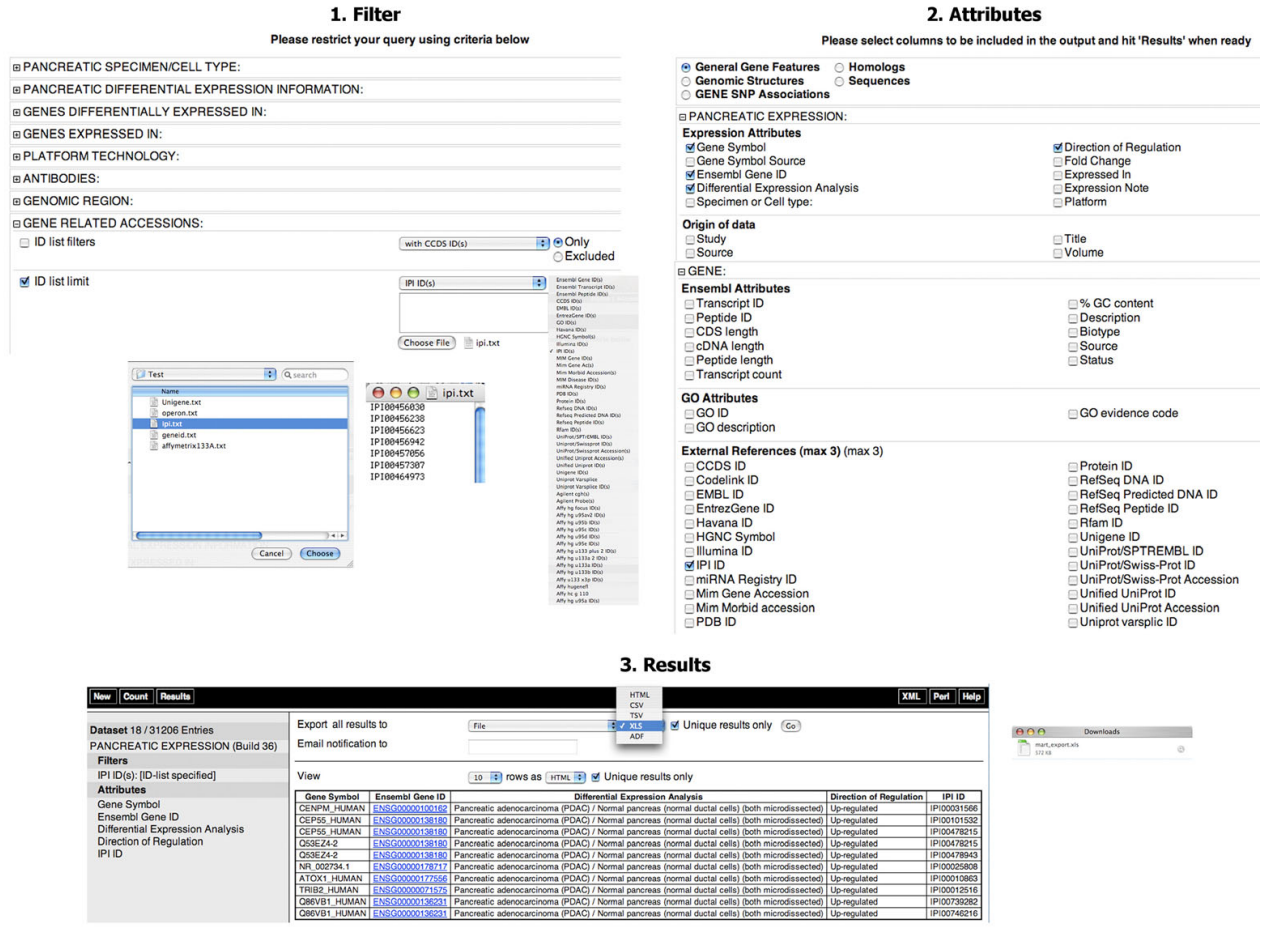
**Meta-analysis of pancreatic cancer data**. In this example, our goal was to retrieve the sets of overlapping genes between user's data obtained by a proteomics platform with IPI identifiers and those reported in the studies stored in the Pancreatic Expression database. 1. Filter: one can restrict the query by clicking on the "*Filters*" node on the left and expanding the filter section "*GENE RELATED ACCESSIONS:*" using the "+" box. Next one can choose the "*ID*" list limit simply by clicking on the left checkbox and selecting the identifiers for the experimental results from the right drop-down menu. In this example, the "*IPI ID(s*)" is used but one can choose any other identifiers such as Uniprot, Affymetrix or Illumina etc. One can copy and paste the list of identifiers in the box or browse to read directly from a file. 2. Attributes: one can click on the "*Attributes*" node on the left and start choosing attributes from the "*General Gene Features*" page. One can expand the "*PANCREATIC EXPRESSION:*" section or other sections and check other attributes to add to the results. 3. Results: for saving the whole result set, one can choose the appropriate action(s) at the top of the page, select the format, for example Excel, and click the "*Go*" button to export the results.

Our database also allows cross-platform meta-analysis. Scientists can investigate pancreatic expression profiling performed across a wide range of different platforms (such as cDNA arrays or oligo arrays) to detect the most consistent sets of de-regulated genes (Figure 7). Importantly, scientists can also retrieve the sets of overlapping genes between their own results obtained by their particular platform (Proteomics, Affymetrix, Illumina etc.) and annotation method (UniProt, RefSeq, HGNC Hugo etc.) and those reported in the studies stored in the Pancreatic Expression database (Figure 8).

**Figure 9:**
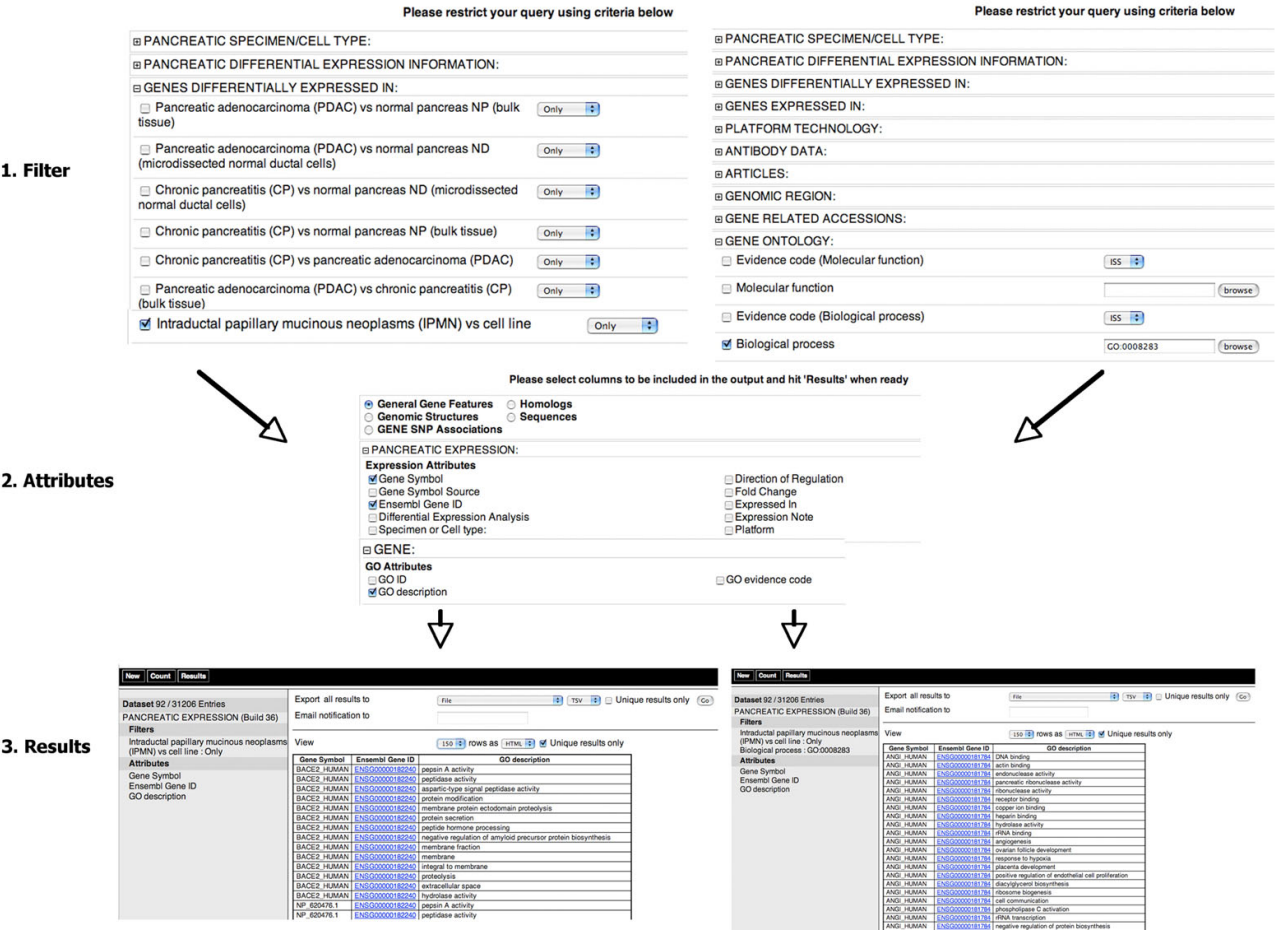
**Ontology for pancreatic cancer genes**. The goal of this example was to obtain the gene ontology classification of the genes differentially expressed in intraductal papillary mucinous neoplasms (IPMN) and/or mine the retrieved genes for cell proliferation process using the ontology term GO:0008283. 1. Filter: one can restrict the query by clicking on the "Filters" node on the left and selecting filters on the right. A. restriction to a cancer subtype comparison, for example by expanding the filter section "*GENES DIFFERENTIALLY EXPRESSED IN:*" and selecting for genes differentially expressed in "*Intraductal papillary mucinous neoplasms (IPMN) vs cell line*" B. restriction to a cancer subtype comparison and cell proliferation biological process "GO:0008283" by expanding the filter section "*GENES DIFFERENTIALLY EXPRESSED IN:*" and selecting for genes differentially expressed in "*Intraductal papillary mucinous neoplasms (IPMN) vs cell line*", expanding the filter section "*GENE ONTOLOGY:*", selecting "*Biological process*" and typing in "*GO:0008283*". 2. Attributes: one can select attributes by clicking on the "*Attributes*" node on the left, choosing the "*General Gene Features*" attribute page, expanding the attribute section "*PANCREATIC EXPRESSION*" to remove "*Differential Expression Analysis*" and "*Direction of Regulation*" default options, expanding the attribute section "*GENE*" and selecting "*GO ID*" under the "*GO Attributes*". 3. Results: one can preview the results by clicking the "*Results*" button on the toolbar. By default this shows the first 10 results for A and B in hyper-linked HTML. The number and format can be altered using the drop downs above this preview.

**Figure 10:**
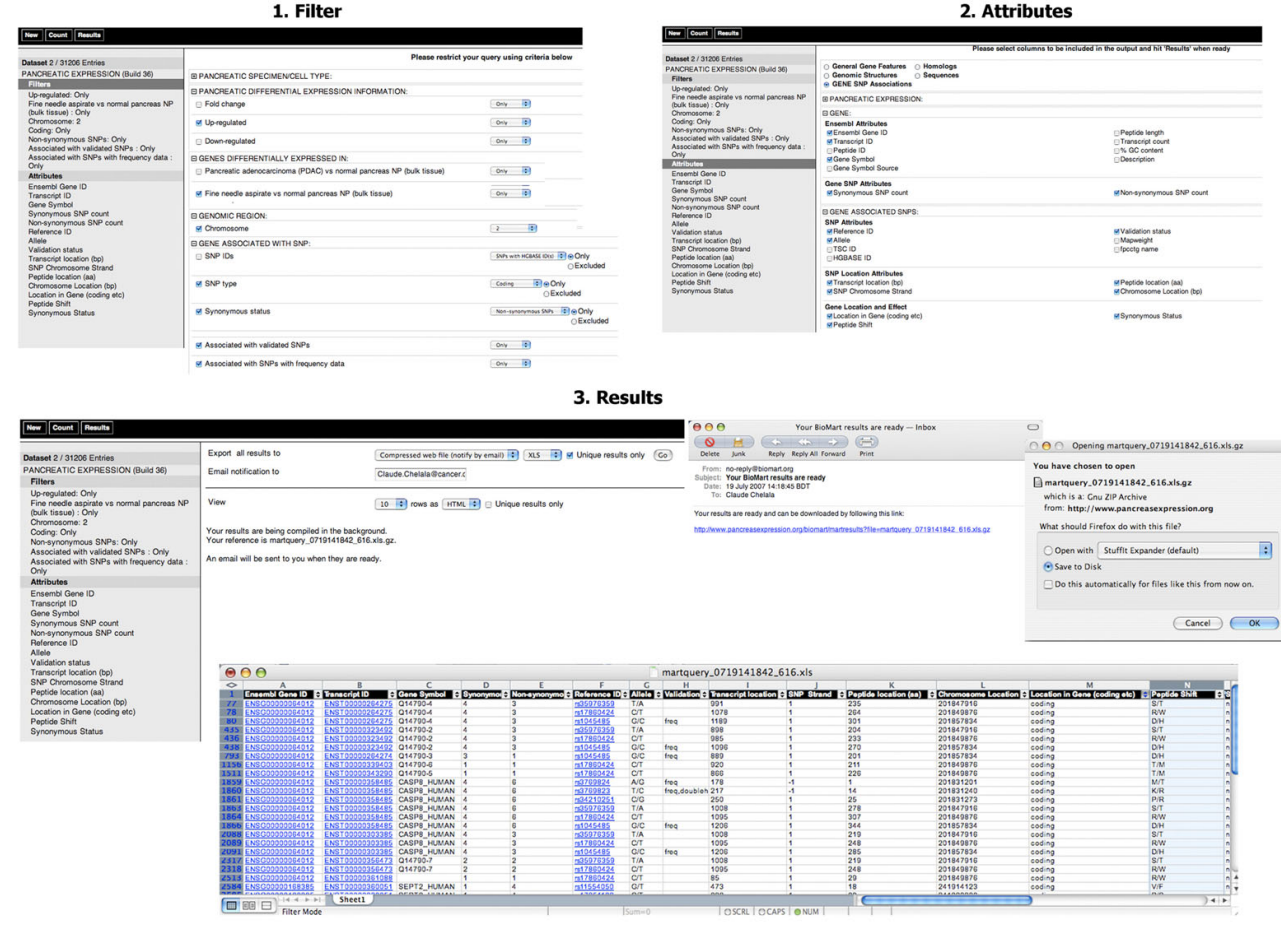
**Design of pancreatic cancer association studies**. The goal of this example was to target SNPs for pancreatic cancer association studies by selecting for coding non-synonymous, validated SNPs with frequency data associated with genes involved in pancreatic cancer. 1. Filter: one can restrict the query by clicking on the "*Filters*" node on the left and selecting "*Up-regulated*" from the filter section "*PANCREATIC DIFFERENTIAL EXPRESSION INFORMATION:*", "*Fine needle aspirate vs normal pancreas NP (bulk tissue)" *from the filter section "*GENES DIFFERENTIALLY EXPRESSED IN:*" and "*Chromosome 2*" from the filter section "*GENOMIC REGION*". For SNP filters, one can expand the filter section "*GENE ASSOCIATED WITH SNP:*", select "*SNP type*" as "coding" from its drop down menu, "*Synonymous status*" as "*Non-synonymous SNPs*" from its drop down menu as well as "*Associated with validated SNPs*" and "*Associated with SNPs with frequency data*". 2. Attributes: one can click on the "*Attributes*" node on the left and start choosing attributes on the right from the "*Gene SNP associations*" attribute page. In this example, one can expand the attribute section "*PANCREATIC EXPRESSION*" to remove "*Differential Expression Analysis*" and "*Direction of Regulation*" default options, expand the attribute section "*GENE*" to select "*Transcript ID*" under the "Ensembl Attributes", "Synonymous SNP count" and "Non-synonymous SNP count" under "Gene SNP Attributes", expand the "*GENE ASSOCIATED SNPS:*" section to select the "*Reference ID*", "*Allele*" and "*Validation status*" under the "*SNP Attributes*", "*Transcript location (bp*)", "*SNP Chromosome Strand*", "*Peptide location (aa*)" and "*Chromosome Location (bp*)" under the "*SNP Location Attributes:"*, and finally, "*Location in Gene (coding etc*)", "*Peptide Shift*" as well a "*Synonymous Status*" under the "*Gene Location and Effect*". 3. Results: one can preview the results by clicking the "*Results*" button on the toolbar. For particularly intensive queries, which is true in this example, one can use the web file options by entering an email address. An email will then be sent to the given address when the results are ready and one can click on the link in the email to download the results. In this example, results are viewed and filtered with Excel.

**Figure 11:**
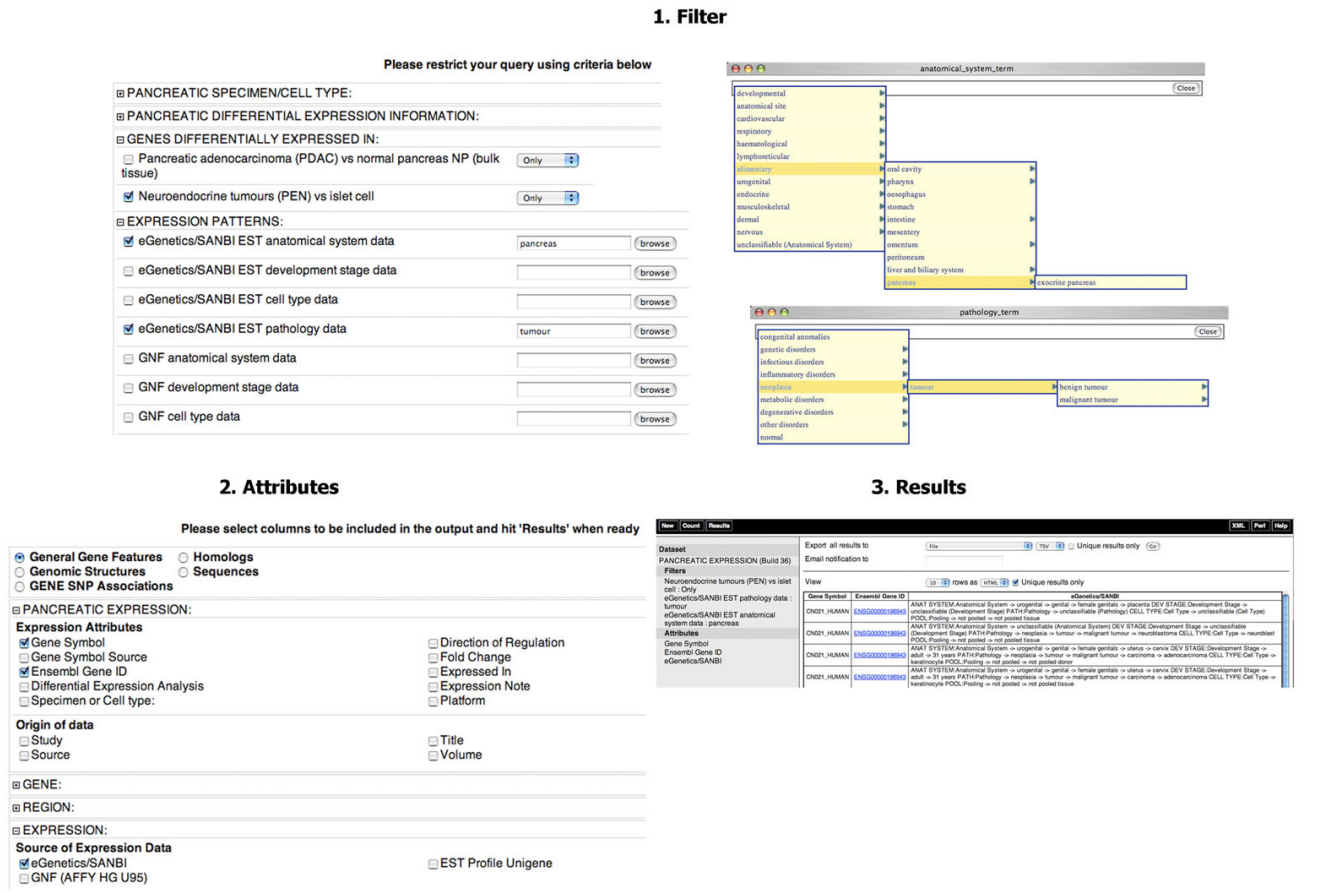
**Pancreatic cancer gene expression**. In this example, our goal was to retrieve additional expression data from a specific anatomical site for genes differentially regulated in neuroendocrine tumours (PEN). 1. Filter: one can restrict the query by clicking on the "*Filters*" node on the left, expanding the filter section "*GENES DIFFERENTIALLY EXPRESSED IN:*" to restricted the query for genes differentially expressed in "*Neuroendocrine tumours (PEN) vs islet cell*". Next, one can expand the filter section "*EXPRESSION PATTERNS:*" and restrict the query further to genes having expression profiles in pancreas and tumour by selecting. "*eGenetics/SANBI EST anatomical system data*" for "*pancreas*" (*eGenetics/SANBI EST anatomical system data -> alimentary -> pancreas*) and the "*eGenetics/SANBI EST pathology data*" for "*tumour*" (*eGenetics/SANBI EST pathology data -> neoplasia -> tumour*). 2. Attributes: one can click on the "*Attributes*" node on the left and start choosing attributes on the right. In this example, one can choose the "*General Gene Features*" attribute page, expand the attribute section "*PANCREATIC EXPRESSION*" to remove "*Differential Expression Analysis*" and "*Direction of Regulation*" options selected by default, expand the attribute section "*EXPRESSION:*" and select the "*eGenetics/SANBI*" under the "*Source of Expression Data"*. 3. Results: one can preview the results by clicking the Results button on the toolbar.

**Figure 12:**
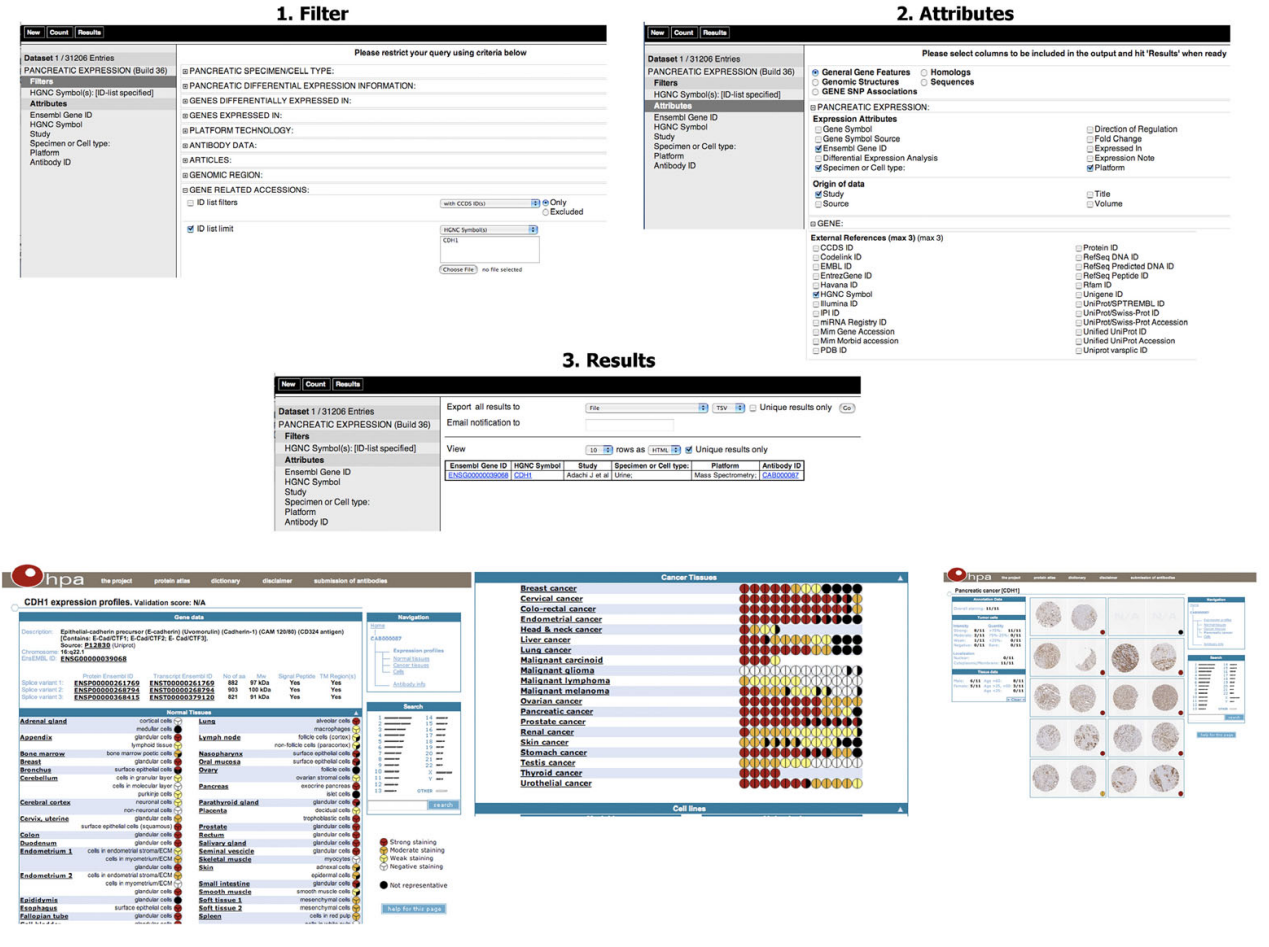
**Immunohistochemistry data**. In this example, the goal was to show how to obtain immunohistochemistry data for the E-cadherin gene. 1. Filter: one can restrict the query by clicking on the "*Filters*" node on the left, expanding the filter section "*GENE RELATED ACCESSIONS:*" using the "+" box, choosing the "*ID*" list limit simply by clicking on the left checkbox and selecting the "*HGNC Symbol(s*)" and typing in the E-cadherin gene symbol "*CDH1*". 2. Attributes: one can click on the "*Attributes*" node on the left and start choosing attributes from the "*General Gene Features*" page. In this example, one can expand the "*PANCREATIC EXPRESSION:*" section, check "*Ensembl Gene ID*", "*Specimen or Cell type*", "*Platform" under *"*Expression Attributes*" and "*Study*" under "*Origin of data*". Once can also expand the "*GENE:*" section to check "*HGNC Symbol*" under the "*External References (max 3*)" and "*ANTIBODIES:*" to check "*Antibody ID*". 3. Results: one can click the "*Results*" button to view the results. The antibody ID for "*CDH1*" is displayed with a hyper-link to the Human Protein Atlas where immunohistochemistry data for normal and cancer tissues, as well as for cell lines, can be displayed.

As they are available through the BiomaRt package [30], annotations can be added to any disease expression profiling experiment, which will allow detection of genes de-regulated in both pancreatic cancer and any other disease (Figure 2B). One can also obtain the gene ontology classification of the retrieved datasets or mine the genes of interest for a specific ontology term (Figure 9). Investigations using association studies can be designed using the Pancreatic Expression database by selecting a specific category of functional consequences (coding non-synonymous, 3' UTR, 5' UTR, splice site etc.) for SNPs associated with genes involved in pancreatic cancer (Figure 10). Expression data from a specific anatomical site can also be retrieved (Figure 11). Researchers can obtain immunohistochemistry data, where available, by selecting the antibodies filter (Figure 12). Scientists interested in promoter analysis can easily combine the gene search with the human genome upstream sequences and therefore collect the promoter sequences in a fast and simple way allowing further analysis of transcription factor-binding sites (Figure 1A).

## Conclusion

Our integration model brings together relevant pancreatic cancer datasets and annotations from public sources and enables scientists to perform a wide variety of complex queries on various types of data. The design of the database allows easy integration of additional modules and annotations from new public databases.

The Pancreatic Expression database constitutes a unique and valuable resource for the wider cancer research community, and is in rapid and constant development. We aim to continuously import new data sources and update the database on a regular basis, and invite scientists worldwide to deposit and share their data.

Although initially constructed using pancreatic cancer expression datasets, we have designed and implemented a generic system that can be easily modified and applied to any other type of cancer. The system is available for collaboration with all interested research groups either by extending it to include other cancer data or by sharing our model should they want to adopt it for their data.

## Availability and requirements

Project name: Pancreatic Expression database

Project home page: 

Operating system(s): Platform independent; Standard WWW browser (Safari, Firefox)

Programming language: Perl, SQL, BioMart data management system

Licence: The database is freely available to academic and non-academic users. However, should you find the Pancreatic Expression database useful to your work, please cite this paper.

## Authors' contributions

CC designed and implemented the web site and database, annotated and integrated the data, contributed to the data collection and wrote the manuscript. SAH defined the pancreatic cancer modules, collected pancreatic data, tested the database, contributed to the revision of the manuscript and continuous discussion. HJW, SB, DH and TPR were involved in the pancreatic data collection. TCJ contributed to the definition of the pancreatic cancer modules and pancreatic data collection. TCJ and NRL provided valuable guidance and expertise on pancreatic cancer, contributed to the critical revision of the manuscript and continuous discussion. All authors read the final manuscript.

## References

[B1] Schneider G, Siveke JT, Eckel F, Schmid RM (2005). Pancreatic cancer: basic and clinical aspects. Gastroenterology.

[B2] Pancreatic Expression database. http://www.pancreasexpression.org.

[B3] MySQL. http://www.mysql.com.

[B4] BioMart. http://www.biomart.org.

[B5] Ensembl. http://www.ensembl.org.

[B6] Ensembl microarray probeset mapping. http://www.ensembl.org/info/about/docs/microarray_probe_set_mapping.html.

[B7] Human Protein Atlas. http://www.proteinatlas.org.

[B8] Van Heek NT, Maitra A, Koopmann J, Fedarko N, Jain A, Rahman A, Iacobuzio-Donahue CA, Adsay V, Ashfaq R, Yeo CJ, Cameron JL, Offerhaus JA, Hruban RH, Berg KD, Goggins M (2004). Gene expression profiling identifies markers of ampullary adenocarcinoma. Cancer Biol Ther.

[B9] Adachi J, Kumar C, Zhang Y, Olsen JV, Mann M (2006). The human urinary proteome contains more than 1500 proteins, including a large proportion of membrane proteins. Genome Biol.

[B10] Anderson NL, Polanski M, Pieper R, Gatlin T, Tirumalai RS, Conrads TP, Veenstra TD, Adkins JN, Pounds JG, Fagan R, Lobley A (2004). The human plasma proteome: a nonredundant list developed by combination of four separate sources. Mol Cell Proteomics.

[B11] Buchholz M, Braun M, Heidenblut A, Kestler HA, Kloppel G, Schmiegel W, Hahn SA, Luttges J, Gress TM (2005). Transcriptome analysis of microdissected pancreatic intraepithelial neoplastic lesions. Oncogene.

[B12] Crnogorac-Jurcevic T, Efthimiou E, Capelli P, Blaveri E, Baron A, Terris B, Jones M, Tyson K, Bassi C, Scarpa A, Lemoine NR (2001). Gene expression profiles of pancreatic cancer and stromal desmoplasia. Oncogene.

[B13] Crnogorac-Jurcevic T, Efthimiou E, Nielsen T, Loader J, Terris B, Stamp G, Baron A, Scarpa A, Lemoine NR (2002). Expression profiling of microdissected pancreatic adenocarcinomas. Oncogene.

[B14] Crnogorac-Jurcevic T, Gangeswaran R, Bhakta V, Capurso G, Lattimore S, Akada M, Sunamura M, Prime W, Campbell F, Brentnall TA, Costello E, Neoptolemos J, Lemoine NR (2005). Proteomic analysis of chronic pancreatitis and pancreatic adenocarcinoma. Gastroenterology.

[B15] Crnogorac-Jurcevic T, Missiaglia E, Blaveri E, Gangeswaran R, Jones M, Terris B, Costello E, Neoptolemos JP, Lemoine NR (2003). Molecular alterations in pancreatic carcinoma: expression profiling shows that dysregulated expression of S100 genes is highly prevalent. J Pathol.

[B16] Friess H, Ding J, Kleeff J, Fenkell L, Rosinski JA, Guweidhi A, Reidhaar-Olson JF, Korc M, Hammer J, Buchler MW (2003). Microarray-based identification of differentially expressed growth- and metastasis-associated genes in pancreatic cancer. Cell Mol Life Sci.

[B17] Grutzmann R, Pilarsky C, Ammerpohl O, Luttges J, Bohme A, Sipos B, Foerder M, Alldinger I, Jahnke B, Schackert HK, Kalthoff H, Kremer B, Kloppel G, Saeger HD (2004). Gene expression profiling of microdissected pancreatic ductal carcinomas using high-density DNA microarrays. Neoplasia.

[B18] Hu L, Evers S, Lu ZH, Shen Y, Chen J (2004). Two-dimensional protein database of human pancreas. Electrophoresis.

[B19] Iacobuzio-Donahue CA, Maitra A, Shen-Ong GL, van Heek T, Ashfaq R, Meyer R, Walter K, Berg K, Hollingsworth MA, Cameron JL, Yeo CJ, Kern SE, Goggins M, Hruban RH (2002). Discovery of novel tumor markers of pancreatic cancer using global gene expression technology. Am J Pathol.

[B20] Logsdon CD, Simeone DM, Binkley C, Arumugam T, Greenson JK, Giordano TJ, Misek DE, Kuick R, Hanash S (2003). Molecular profiling of pancreatic adenocarcinoma and chronic pancreatitis identifies multiple genes differentially regulated in pancreatic cancer. Cancer Res.

[B21] Lu Z, Hu L, Evers S, Chen J, Shen Y (2004). Differential expression profiling of human pancreatic adenocarcinoma and healthy pancreatic tissue. Proteomics.

[B22] Maitra A, Hansel DE, Argani P, Ashfaq R, Rahman A, Naji A, Deng S, Geradts J, Hawthorne L, House MG, Yeo CJ (2003). Global expression analysis of well-differentiated pancreatic endocrine neoplasms using oligonucleotide microarrays. Clin Cancer Res.

[B23] Nakamura T, Furukawa Y, Nakagawa H, Tsunoda T, Ohigashi H, Murata K, Ishikawa O, Ohgaki K, Kashimura N, Miyamoto M, Hirano S, Kondo S, Katoh H, Nakamura Y, Katagiri T (2004). Genome-wide cDNA microarray analysis of gene expression profiles in pancreatic cancers using populations of tumor cells and normal ductal epithelial cells selected for purity by laser microdissection. Oncogene.

[B24] Segara D, Biankin AV, Kench JG, Langusch CC, Dawson AC, Skalicky DA, Gotley DC, Coleman MJ, Sutherland RL, Henshall SM (2005). Expression of HOXB2, a retinoic acid signaling target in pancreatic cancer and pancreatic intraepithelial neoplasia. Clin Cancer Res.

[B25] Shen J, Person MD, Zhu J, Abbruzzese JL, Li D (2004). Protein expression profiles in pancreatic adenocarcinoma compared with normal pancreatic tissue and tissue affected by pancreatitis as detected by two-dimensional gel electrophoresis and mass spectrometry. Cancer Res.

[B26] Tan ZJ, Hu XG, Cao GS, Tang Y (2003). Analysis of gene expression profile of pancreatic carcinoma using cDNA microarray. World J Gastroenterol.

[B27] Terris B, Blaveri E, Crnogorac-Jurcevic T, Jones M, Missiaglia E, Ruszniewski P, Sauvanet A, Lemoine NR (2002). Characterization of gene expression profiles in intraductal papillary-mucinous tumors of the pancreas. Am J Pathol.

[B28] Pancreatic Expression database web-based query interface. http://www.pancreasexpression.org/biomart/martview.

[B29] Bioconductor. http://www.bioconductor.org.

[B30] Durinck S, Moreau Y, Kasprzyk A, Davis S, De Moor B, Brazma A, Huber W (2005). BioMart and Bioconductor: a powerful link between biological databases and microarray data analysis. Bioinformatics.

[B31] R project. http://www.r-project.org.

[B32] Hull D, Wolstencroft K, Stevens R, Goble C, Pocock MR, Li P, Oinn T (2006). Taverna: a tool for building and running workflows of services. Nucleic Acids Res.

[B33] Galaxy. http://main.g2.bx.psu.edu.

[B34] Pancreatic Expression database access through web services. http://www.pancreasexpression.org/biomart/martservice.

